# Predicting epidemics using search engine data: a comparative study on measles in the largest countries of Europe

**DOI:** 10.1186/s12889-020-10106-8

**Published:** 2021-01-21

**Authors:** Loukas Samaras, Miguel-Angel Sicilia, Elena García-Barriocanal

**Affiliations:** Computer Science Department, Polytechnic Building, University of Alcalá, Ctra. De Barcelona km. 33.6, 28871 Alcalá de Henares (Madrid), Spain

**Keywords:** Syndromic surveillance, Measles, Linear regression, Forecasting, Programming languages, Computational science

## Abstract

**Background:**

In recent years new forms of syndromic surveillance that use data from the Internet have been proposed. These have been developed to assist the early prediction of epidemics in various cases and diseases. It has been found that these systems are accurate in monitoring and predicting outbreaks before these are observed in population and, therefore, they can be used as a complement to other methods. In this research, our aim is to examine a highly infectious disease, measles, as there is no extensive literature on forecasting measles using Internet data,

**Methods:**

This research has been conducted with official data on measles for 5 years (2013–2018) from the competent authority of the European Union (European Center of Disease and Prevention - ECDC) and data obtained from Google Trends by using scripts coded in Python. We compared regression models forecasting the development of measles in the five countries.

**Results:**

Results show that measles can be estimated and predicted through Google Trends in terms of time, volume and the overall spread. The combined results reveal a strong relationship of measles cases with the predicted cases (correlation coefficient *R*= 0.779 in two-tailed significance *p*< 0.01). The mean standard error was relatively low 45.2 (12.19%) for the combined results. However, major differences and deviations were observed for countries with a relatively low impact of measles, such as the United Kingdom and Spain. For these countries, alternative models were tested in an attempt to improve the results.

**Conclusions:**

The estimation of measles cases from Google Trends produces acceptable results and can help predict outbreaks in a robust and sound manner, at least 2 months in advance. Python scripts can be used individually or within the framework of an integrated Internet surveillance system for tracking epidemics as the one addressed here.

## Background

Syndromic surveillance is concerned with the monitoring of emerging infectious diseases. It is a key element in public health, in order to track, estimate, predict and prevent the threats of prevalent outbreaks among population for various infections. Novel tools use Web-based methods and Internet data for major infections that can be transmitted from person to person in a place or because of various factors of modern way of living, such as frequent travelling or events gathering large numbers of individuals.

Although medical science has advanced regarding treatment, pathogen discovery or diagnostics, infections can be widely spread and cause severe health problems. On the other hand, modern technology has introduced new methods and tools. From 2004 [1], a steady use of Internet-surveillance systems can be observed. Rees et al. (2019) [2] describe this new form of surveillance as *a new generation of surveillance strategies.* Christaki (2015) [3] analyses modern trends of Internet-based surveillance, taking advantage of computational science methods. Web queries, Google Trends [4], Google Flu Trends [5], event-based surveillance, remote sensing technology, social media communications and mobile phones are examples of the new trend of using technology to supplement other methods of monitoring and alerting about infections and outbreaks. Until today, various diseases have been tested using those approaches, including influenza, Ebola virus, HIV/ AIDS, Dengue fever and many others.

Measles is a case that has not been extensively examined in relation to Internet surveillance, although its spread is considered as quite substantial and its implications are also severe. According to the World Health Organization (WHO) [6], measles is considered one of the most infectious diseases and can cause a great number of cases, as well as deaths. While measles mainly affects children under 15 years of age, the transmission can be also be spread in adults. WHO’s 2000 estimation of children deaths was 535,000 worldwide. Most of them occurred in developing countries and this burden accounted for 5% of all under-five mortality [7]. WHO reveals that prior to the availability of measles vaccine, measles infected over 90% of children before they reached the age of 15. These infections resulted in more than two million deaths and between 15,000 and 60,000 cases of blindness annually worldwide.

Although WHO recognizes that measles continues to be a problem in developing countries, in the United States (US) the implications are also important. The US Centers for Disease Control and Prevention (CDC) estimate in their latest report [8] that during the pre-vaccine era (before 1963), each year measles caused 3 to 4 million cases, of which 500,000 were reported to CDC. In modern years though, many cases are observed in the US. Only in 2016, over 650 cases are officially reported. The CDC final estimation is that these cases result in hospitalization for 25% of cases, encephalitis (inflammation of the brain) for 0.1% and death to 0.1–0.2% of the cases. Despite the significant progress of medicine and vaccination, during the last years, CDC believe that measles remains a leading cause of vaccine-preventable infant mortality.

The cost burden of measles treatment and hospitalization also remains high. WHO states that for measles and rubella the annual cost estimation in 2004 confirmed continued cost-savings associated with the current US 2-dose Measles, Mumps, and Rubella (MMR) routine vaccination schedule and estimated annual net benefits exceeding US$ 9.7 billion [9]. For US alone, WHO estimated a net saving for the WHO Region of the Americas of over US$ 282 million (US$2011), just for the year 2011. CDC estimate in their latest report that, for 107 cases in 2011, the number of cases (outbreaks) resulted to a public financial burden of 2.7–5.3 million US$.

In the European Union/ European Economic Area (EU/EEA), the European Centre for Disease Prevention and Control (ECDC) [10] recorded 36 deaths during the last 3 years. While measles recorded a high outbreak in the EU/EEA during the years of 2010–2011 with an average of 30,000 cases in all 30 countries, from 2012 until 2017 the measles activity was relatively low. However, the infection activity started to rise from 2017 and over 10,000 cases are annually reported since that year.

Google Trends have been used to a large extend to track epidemics. Along with social media (e.g., Twitter), it is the most common and easy to use data source. During the last 3 years, Google Trends were used to predict syphilis (2018) [11], respiratory syncytial virus (2018) [12] and vaccination (2017) [13]. The last research is a direct reference to vaccines, but indirectly relates them to infectious diseases. Furthermore, forecasting methods were used for Dengue fever (2016) [14] and AIDS (2018) [15]. For measles, a recent study [16] examines the usefulness of Facebook and Twitter posts (2017) to the consistent social media engagement by individuals expressing vaccine hesitancy, contrasted with media. The researchers of the study conclude that the association of social media and vaccination may result from more consistent social media engagement by individuals expressing vaccine hesitancy, contrasted with media- or event-driven episodic interest on the part of individuals favoring current policy.

Considering all the above, the scope of this study is:

- To assess the feasibility to predict measles using Google Trends data.
- To contribute to the research of the Internet surveillance systems on measles.
- To evaluate prediction methods and techniques, based on specific criteria.
- To improve the accuracy of prediction for countries with a low impact of measles, by using additional techniques

The rest of this paper is structured as follows, Section “Methods” describes the data and methods used. In Section “Results”, results and analysis are provided and finally, in Sections “Discussion” and “Conclusions” we provide the discussion and the main conclusions on this work respectively.

## Methods

The research was conducted in three stages: specification of research questions and evaluating criteria, data acquisition and finally, data analysis, interpretation of the results and conclusions. Specifically, we wanted to test whether a linear regression model can be applied or not and in which cases provides the ability to track and predict measles in the countries of Europe with the largest population. Based on the abovementioned aims of this research, the research questions and the evaluation criteria were set as described in Table 1. These questions identify the major aspects of our analysis and help in determining the criteria to evaluate the current methodology and the proper model to use. It is also significant to notice that for the last criterion, the desired value must be lower than 28%, relative to each country.

**Table 1:** Research questions and Evaluation Criteria

no	Research Question (RQ)	Evaluation Criteria (C)
**1**	The predicted cases show a strong correlation with the real cases	c1-Correlation: The correlation between measles cases and predicted cases must exceed 0.650 and must also be statistically significant. The significance level (*p*) was set to be < 0.01 in two-tailed significance
**2**	The prediction shows the right time of measles’ outbreaks	c2-Time: The prediction point of measles outbreaks must not exceed one month in relation to the real cases and must not be observed after the outbreak
**3**	The predicted value of outbreaks is close to the real cases	c3-Volume of predicted cases during outbreak periods: must not exceed 28% of the real cases during outbreaks
**4**	The prediction includes all periods with excessive activity of measles (outbreaks)	c4-Outbreak predicted periods: the distributions of each prediction must include all outbreak periods within the 5-year examined period
**5**	The error of the estimate (MSE%) is smaller than 28%	c5-Mean Standard Error (MSE): the MSE of all predicted cases must not exceed 28% of the real cases mean

As described below, we used linear regression over the data for all the countries. However, some countries required testing alternative methods to achieve a better quality.

The rest of this section describes the data used (“Data sources”), and the linear regression used as a model (“Linear regression”). Then, in subsection “Model enchancement testing”, the additional modeling used for the above-mentioned countries that required an alternative is described.

### Data sources

We used two data sets. The first comes from the ECDC official web site, which publishes monthly reports on measles. ECDC monitors 30 countries of the EU/EEA and publishes reports with cases of measles each month. Each report includes data with a 2 months lag, e.g. the report of October 2018 has information and data until August 2018.

We created time series of the 5 years from October 2013 to August 2018 (59 months). We selected the countries with the most population of Europe because we noticed that for other countries there were a high number of zero cases or missing data. Therefore, we decided to use data from Italy, France, Germany, the UK and Spain. These five countries represent 323,256,460 people [17], 62.30% of the total population of the EU/EEA, nearly the population of the US, while the aggregation of measles cases in these countries are 21,015, meaning the 53.72% of the total cases in all countries.

The second data set was from Google Trends for the same period. Google Trends publishes the search volume on terms that are submitted to Google search engine for one or more specific keywords. As Google Trends state, Google Trends data is an unbiased sample of Google search data and only a percentage of searches are used to compile trends data [18]. The data sources of Google Trends are derived from real-time data from the previous 7 days (weekly data) and non-realtime data coming back from 2004 on monthly basis. This search volume is scaled from 0 to 100 and, on the web platform, data up to five past years are shown weekly, while data from 2004 are presented monthly. The values of this scale of 0–100 may be sometimes revised, adjusted and corrected by Google. Nevertheless, the differences are not so large and would cause no different results. Google explain the overall adjustment procedure and how this scale 0–100 is produced [19]. Since we did not use the web application of Google, but Python scripts, we managed to acquire daily data and then we aggregated them on monthly basis to match up with data on measles.

*Python* is a flexible programming language often used in these kinds of studies, e.g. in a recent one (2017) scientists discussed and analyzed Python techniques in combination with Google Search Trends to test three cases [20]. To capture the data from Google, we used *Python* scripts by applying *Pytrends* [21]. It uses the localization system of the *ISO 3166-1 alpha-2* standard [22]. We used the term *measles* in each of the five languages of the five countries under examination. These are the following: *morbillo* for Italy, *rougeole* for France, *Masern* for Germany, *measles* for the UK and *sarampion* for Spain. Initially, we used the term *sarampión* for Spain, but the extracted dataset had too many zeros, which would made estimation models unfeasible*.*

### Linear regression

We performed single-parameter linear regressions for each country using IBM SPSS v.23.0.0.0 64-bit. The Simple Linear Regression model (SLR) is a statistical method that allows summarizing and studying of the relationships between two continuous (quantitative) variables [23]. The dependent variable in our model was the measles cases and the independent variable (predictor) is Google data, as in the following equation:

1 $$ {\mathrm{Y}}_{\mathrm{t}}={\mathrm{b}}_0+{\mathrm{b}}_1{\mathrm{X}}_{\mathrm{t}}+{\mathrm{e}}_{\mathrm{t}} $$

where **Y**_**t**_ =the value of measles cases, **b**_**0**_ is the constant of the model, **b**_**1**_ is the model parameter and **X**_**t**_ is the independent value of Google search volume (predictor). The value **e**_**t**_ stands for the error of the estimate and we assume that **e**_**t**_ **~ N (**0, σ^2^**)**.

As far as the Mean Standard Error and Mean Square Error are concerned, there has been an argument since many years regarding which metric provides the best accuracy of prediction. For example, RA Fisher (1920) [24] believes that, generally, the second can be better, but sometimes the standard error may be more accurate. In fact, both can assist the correlation procedure, while the square error can be better when it comes to normal distributions or variance analysis. Temperature forecasting has a scale that may be resemble to the normal probability curve and it is similar all over the world. (Allan H. Murphy (1988) [25]. In this case, the authors conducted a research by using the square error. On the other hand, square error is a larger number than the standard error, which may misjudge estimations when we examine and compare different scales (e.g., measles in different countries) or small data samples and, especially, when their distributions are not alike the normal distribution. Finally, the standard error has the ability to adapt to the size of the sample because of the square root of sample cases, which is included in the denominator of the equation. In this way, it becomes ideal for small or very small samples. We have, therefore, chosen to use the Mean Standard Error in the case of measles.

The linearity of regression and homoscedasticity was tested looking at residuals versus predicted values (regression standardized predicted values versus standardized residuals with *Line – Loess Fit* curve). The normality of residuals was diagnosed using histograms and Normal P-P plots of standardized residuals, Normal Q-Q Plots of standardized residuals with Line – Loess Fit curve, and Kolmogorov-Smirnov and Shapiro-Wilk tests for residuals.

The time trend of residuals was examined using scatterplots of residuals versus order. The prediction for each country was summarized using a comparative table for each country with the metrics defined as evaluation criteria.

### Model enhancement testing

Based on the normal *P-P* plots for the standard residuals, the derived forecasted values and the Pearson *R* correlation coefficients, we checked the produced distributions and we noticed that for the UK and Spain the data looked very different and this was significantly indicated by the residuals’ plots. Since the linear model for the UK and Spain seemed not to show good results, we investigated the feasibility of determining the real values of them by constructing an approximate model. This was based both on the predicted values of the linear model for these two countries and the predicted values of the other three countries, which showed to be more accurate. Therefore, we used a comparative technique to estimate the measles cases of the two countries by combining and weighting both data from each country (UK and Spain) and data from the three other countries, i.e., Italy, France and Germany. Our goal was to try to predict measles of the UK and Spain by using the above combination and finally find out whether this approximation would improve the results or not. This procedure is described as follows:

First, by using linear regressions, we calculated the predicted (expected) values for Italy (**E (1)**_**t**_), France (**E(2)**_**t**_) Germany (**E(3**)_t_), the UK (**E(4)**_**t**_) and Spain (**E(5)**_**t**_).

We then aggregated values for each month. The aggregated variable for each month (t) is named E (sum). The same was done for the Google values with a new variable G (sum)_t_. This is shown in the following equations:

2 $$ \mathrm{E}{\left(\mathrm{sum}\right)}_{\mathrm{t}}=\mathrm{E}{(1)}_{\mathrm{t}}+\mathrm{E}{(2)}_{\mathrm{t}}+\mathrm{E}{(3)}_{\mathrm{t}} $$

3 $$ \mathrm{G}{\left(\mathrm{sum}\right)}_{\mathrm{t}}=\mathrm{G}{(1)}_{\mathrm{t}}+\mathrm{G}{(2)}_{\mathrm{t}}+\mathrm{G}{(3)}_{\mathrm{t}} $$

**E(1)**_**t**_, **E(2)**_**t**_ and **E(3)**_**t**_ are the predicted values of measles cases, derived from the linear regression model for each of the three first countries for every month (t) from t=1 to 59. Respectively, **G(1)**_**t**_, **G(2)**_**t**_ and **G(3)**_**t**_ are the measured values of Google for each of the three countries for each month t=1 to 59

With the following step, we performed a second estimation for the UK and Spain. The rationale was to increase the initially predicted values of the UK and Spain, because they were too low, since the measles activity is relatively low for these countries and for the given period. This fact resulted to very low linear regression predictions. This was achieved by increasing the values from Google by using the following equations:

4 $$ {E}_2{(UK)}_t=G{(UK)}_t\frac{\sum \limits_{i=1}^tY\prime {(IT)}_i+\sum \limits_{i=1}^tY\prime {(FR)}_i+\sum \limits_{i=1}^tY\prime {(DE)}_i}{\sum \limits_{i=1}^tG{(UK)}_i}=G{(UK)}_t\frac{E{(sum)}_i}{\sum \limits_{i=1}^tG{(UK)}_i} $$

5 $$ {E}_2{(ES)}_t=G{(ES)}_t\frac{\sum \limits_{i=1}^tY\prime {(IT)}_i+\sum \limits_{i=1}^tY\prime {(FR)}_i+\sum \limits_{i=1}^tY\prime {(DE)}_i}{\sum \limits_{i=1}^tG{(ES)}_i}=G{(ES)}_t\frac{E{(sum)}_i}{\sum \limits_{i=1}^tG{(ES)}_i} $$

Where:

E_2_(UK)_t_ and E_2_(ES)_t_ are the new expected values of measles for the UK and Spain respectively (for every month t=1 to *n*=59),

G (UK)_t_ and G (ES)_t_ is the values of Google for the UK and Spain respectively for every month t=1 to 59,

$\sum \limits_{i=1}^tG{(UK)}_i$and $\sum \limits_{i=1}^tG{(ES)}_i$ stand for the aggregated Google values from the beginning of the time series until month t for the UK and Spain.

$\sum \limits_{i=1}^tY\prime {\left(\mathrm{IT}\right)}_i$ represents the aggregated predicted values of measles for Italy, which were calculated by the linear regression models, meaning the aggregation from the first month until the current month t and analogously for the rest two countries (FR, DE).

At the last step, the final predictions (E_3_)_t_ for the UK and Spain for every month (t) were determined by combining the initial estimations based on linear regressions and the intermediate estimations, as shown in the following equations:

6 $$ {E}_3{(UK)}_t=\frac{2\ast {E}_1{(UK)}_t+3\ast {E}_2{(UK)}_t}{5} $$

7 $$ {E}_3{(ES)}_t=\frac{2\ast {E}_1{(ES)}_t+3\ast {E}_2{(ES)}_t}{5} $$

As it is shown from the above last equations, both estimations are used weighted by 2 for the initial regressions and by 3 for the intermediate estimations. As it is obvious, we do not use equal weighting between the Google data of the three first and the last two countries, since this way the model produces better precision. By using this technique, the overall improvement of accuracy (correlation) is about 5% for the UK and 6% for Spain. The analogy of two was determined after we tried some different values in order to improve the overall correlation coefficients for these countries. The values that were tested are provided in following Table 2.

**Table 2:** Testing values for correlation improvement

no	E_**1**_	E_**2**_	Improvement (UK)	Improvement (ES)
1	1	4	−0.30%	−0.32%
2	2	3	−1.21%	−1.05%
3	1	1	0%	0%
4	3	2	5.12%	6.04%
5	4	1	1.20%	1.33%

For these countries, we also applied a three-parameter Auto-Regressive Integrated Moving Average model with exogenous variable (ARIMAX (p, q, d)). We applied this model for the cases of the UK and Spain to find out whether a better estimation can be achieved or not. We then compared the predicted cases of this model to the ones from the regression, as well as the errors (MSE) of these two models.

In our case, we applied a model which includes the parameters for lag 0 of the Google data. The model that it was finally used was an ARIMAX (0,1,0), as shown in the following equation:

8 $$ {\mathrm{Y}}_{\mathrm{t}}={\mathrm{Y}}_{\mathrm{t}\hbox{-} 1}+\mathrm{b}\left({\mathrm{X}}_{\mathrm{t}}\hbox{-} {\mathrm{X}}_{\mathrm{t}\hbox{-} 1}\right)+{\mathrm{e}}_{\mathrm{t},}\left\{\mathrm{t}=1,2\dots \kern0.5em 59\right\} $$

where **Y**_**t**_ stands for the values of measles for the current week (t), **Y**_**t-1**_ represents the measles values of the previous week, **b** is the model parameter, **X**_t_, **X**_**t-1**_ are the predictors (values from Google) for every current and previous week (t and t-1) and **e**_**t**_, is the error of the estimate.

## Results

### Combined results

**Fig. 1:**
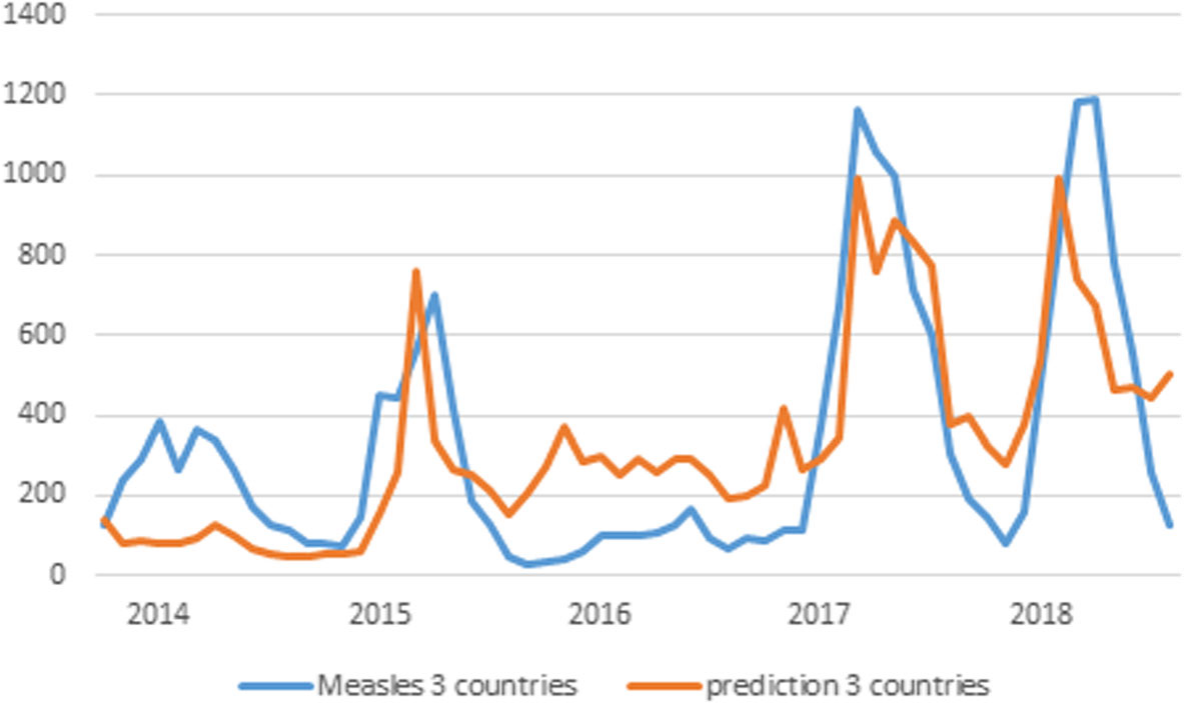
Combined results graph for Italy, Germany and France

**Fig. 2:**
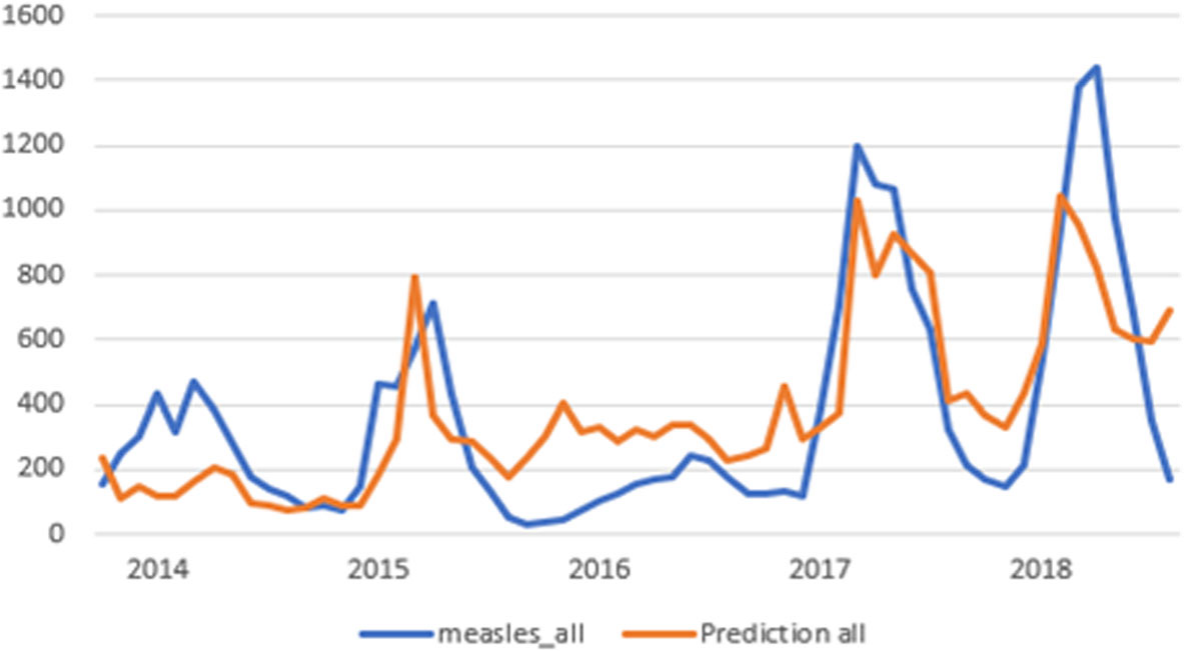
Combined results graph for all countries

Aggregating all predictions, we produced two combined results, as shown in Figs. 1 and 2.

In Fig. 1, the vertical axis represents cases of measles and the horizontal the time (years). Blue line shows real cases and red line shows predicted cases. From this figure we can see that the correlation (coefficient *R* =0.757) is strong at level of significance *p*< 0.01 (two-tailed, *p*=4.0224E-12) and the Mean Standard Error (MSE) is 16.74 (17.18%). Similar results with greater correlation, but also with larger deviations are found for the combination of all five countries, as shown in Fig. 2.

Figure 2 shows all countries, including the UK and Spain. Evaluating the distribution shown in the above figure, in reference to the above-mentioned criteria, we observe the following:

i) **Correlation and significance:** The correlation coefficient is relatively high 0.779 and is significant at the level *p*< 0.01 (two-tailed, sig= 3.626E-13).
ii) **Time accuracy**: There are three periods with a measles outbreak: in the first quarter of the year 2015, in the first courter of 2017 and at the same period of 2018. In more detail, the predicted outbreak of 2015 is shown in March, while the actual one occurs in April. For the year 2017, the predicted period coincides with the true outbreak in March. For the last year of 2018, the predicted outbreak is shown 1 month earlier in March, instead of April. Year 2013 has no severe measles outbreak for the three examined months and, during the year 2014, the activity of measles is close to the monthly five-year average (370.5). This may be the reason that it is not predicted. We, indeed, noticed that this occurs sometimes when the true cases are close to the monthly average of the entire time series or of a single year. Nevertheless, it is not important since it is near the mean of the cases.
iii) **Precision of high values:** For each year, the results are shown in Table 3. As shown in the table, regarding outbreaks, the observed estimated difference (%) lays at the level of 11.86–27.68%, which may be considered as acceptable.
iv) **Prediction of all high values**: As described above, the predicted distribution includes all years which show significant outbreak of measles; 2015, 2017 and 2018. During the years 2013, 2014 and 2016 the measles activity and impact are low, and no significant outbreak is noticed.
v) **Mean Standard Error**: It is relatively small and is observed 12.19%. for all months. Therefore, we consider the prediction as good and we can safely use it.

**Table 3:** Real and predicted outbreaks of measles

Year	Real value	Predicted value	Difference	Difference (%)
2015	710	794	84	11.86%
2017	1194	1030	−164	13.72%
2018	1438	1040	− 398	27.68%
All years	3342	2864	− 478	14.29%

### Results for each country and comparison

**Fig. 3:**
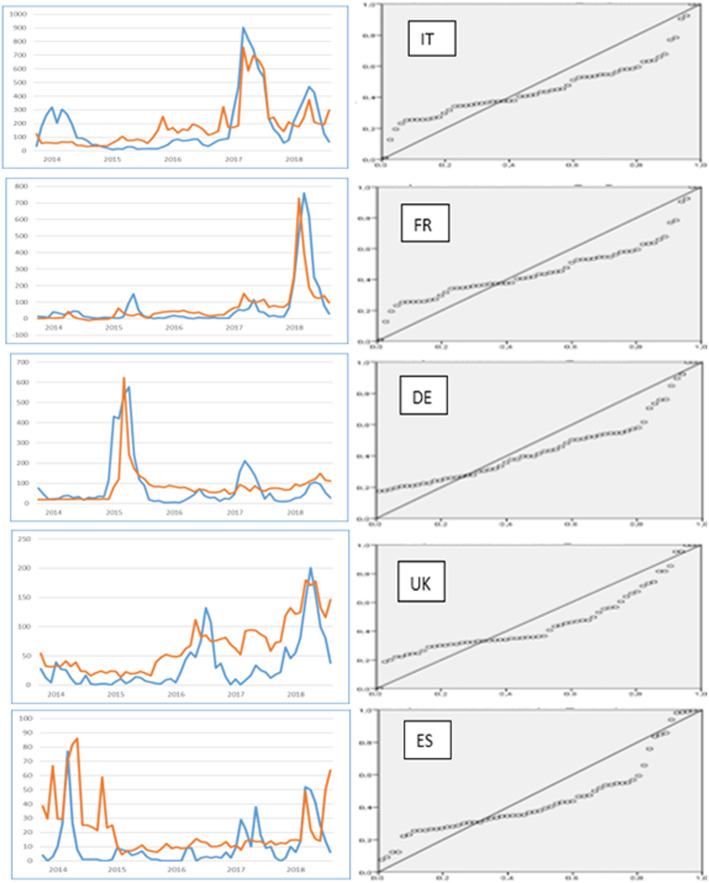
Real cases, Predictions and normal P-P Plots for each country

In this part of our research, we examined and compared the similarities and differences for each country. To better understand the results, we provide graphs of the distributions of real and predicted measles for each country, but also the standard residuals plots, produced by the regression procedure (Fig. 3). On the left side the comparison of true (blue line) and predicted cases (red line) and on the right, the normal P-P plots for residuals.

Measles in Italy has a very high activity in the first quarter of 2017 and a smaller in the same quarter of 2018. As shown in the above figure, the predicted cases are very close to the true cases and at the right time. In 2017, Italy faced a very severe outbreak, after a relative medium activity in the first quarter of 2014. The impact of measles infection resulted to over 900 cases in March of that year. The prediction model provides a good estimation, although with a slight lower volume of cases. The correlation coefficient between measles cases and predicted cases is 0.806 and it is statistically significant. The mean error of the estimate (MSE), expressed as a percentage, was estimated as 11.80%. The mean difference is very low at 0.80% (predicted mean 180.2 versus 179.6 of the real cases).

France has a different measles development. The most cases are observed in 2018 but to a smaller extend of 760 cases during March of 2018. The prediction of this outbreak is also good at 729 cases, predicted 1 month earlier. The correlation is also high in this model with the *R* coefficient to be measured as 0.779. The mean standard error of the estimate is calculated as 21.88%, but the mean of the cases is very close to the real ones (67.11, instead of 66.86) with a very small mean difference of 0.37%.

In Germany, the largest measles infection is observed in the first quarter of 2015, peaked to 577 cases. The predicted value is 622.72 with a difference of − 4.05%, observed 1 month earlier in March of that year. The correlation of all values is statistically significant, and it is 0.676. The mean standard error is found to be 17.86%, while the mean of the predicted cases is the same as for the real cases (81.2) with no difference at all.

Looking at the graphs and the standard residual plots of the three first countries, we found some major differences with the other two (UK and Spain). Both UK and Spain have too low peaks of measles outbreaks, related to the first three countries. UK has a maximum of 132 cases observed in the middle of 2016 (July), considering that the population of the UK is close to Italy and France and it is 20–25% smaller than Germany. Spain has even lower measles cases. In March of 2014, the highest activity of measles is observed with 77 cases. The monthly average is much lower (10.36 cases). Furthermore, the normalized residual plot reveals another difference. In the upper right corner of the plots for the UK and Spain, we can see that the residuals cross the diagonal line from the right side to over this line towards the left.

These observations are the reason why we performed additional research (the methods are described in Section “Methods”) to establish prediction patterns for these two countries. By doing so, we managed to achieve significant correlations at level < 0.01 (two-tailed), but this was not enough, especially for the case of Spain.

The UK has its measles outbreak at the same period of France, i.e. in the first quarter of 2018, which is 201 cases. In addition, a relatively high value of measles (for the UK, not for Europe) is also observed in the middle of 2016 which represents 132 cases. The prediction model has a correlation coefficient of 0.781, as much high as for the previous countries, and it manages to predict the outbreak of 2018. On the other hand, the lower measles activity is not captured well since it is observed 2 months earlier. The difference between real and predicted peak for this period is not high (111.6 cases predicted related to the actual 132 cases). The difference expressed as percentage is − 12.12% and corresponds to only 20.4 cases. The mean standard error is not excessively high (26.90%), although higher, related to the first three countries, but the mean error is significant (95.79%) when it predicts a (monthly) mean of 65.6 cases instead of 33.5 real cases.

Finally, for Spain, the prediction model was not good enough, even though it predicts both the 2014 and 2018 high values of measles. The correlation coefficient *R* is significant, but very low (0.322). Nevertheless, the maximum values are predicted almost well. For the 2014, the prediction shows 86.07, while the real cases are 77. The difference is − 11.8 (− 15.31%) that may be considered as acceptable, but the prediction is shown 2 months later in May of 2014, which cannot be considered as acceptable. In March of 2018, the high values are respectively 48.95 and 52 and the difference is very low (− 5.87%). For this period, the prediction is almost perfect and at the right time. However, the MSE for all years is too high (151.60%). Additionally, before or between these two periods, additional predicted high values are observed.

In the following Table 4, we summarize the results for each county and for the combined values.

**Table 4:** The summary of results (monthly values)

country	Total cases	Cases mean	predicted cases	predicted mean	mean df	Mean df (%)	R	Mean standard error	Mean standard error %
all	21,861	370.5	22,542	382.1	10.3	3,12%	0.779	45.2	12.19%
Italy	10,538	178.6	10,630	180.2	1.6	0,87%	0.806	21.1	11.80%
France	3945	66.9	3960	67.1	0,2	0,37%	0.780	14.6	21.88%
Germany	4791	81.2	4791	81.2	0,0	0,00%	0.676	14.5	17.86%
UK	1976	33.5	3869	65.6	0,0	95.79%	0.781	9.0	26.90%
Spain	611	10.4	1264	21.4	8.5	106.87%	0.322	15.7	151.60%

### Diagnostic results

#### Linearity and homoscedasticity

**Fig. 4:**
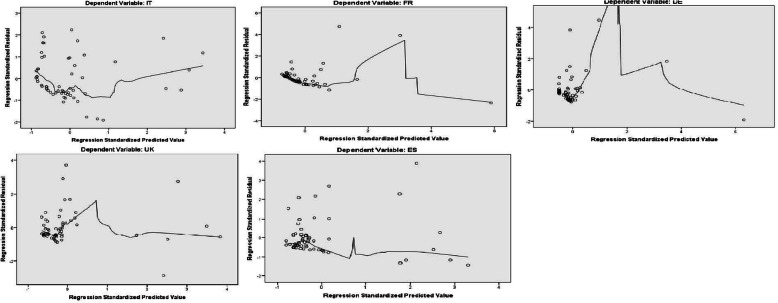
Scatterplots for residuals against predicted values

This is tested by viewing the standardized predicted values against the standardized residuals. This was done with the use of the scatterplots, which also include the *Fit Line – Loess*. This line identically crosses between the center of the observed residuals. In the graph below (Fig. 4), we can see the scatterplots for all countries. It is shown that all values cluster around zero (vertical and horizontal axis), which is the case for the linearity of the model. However, there are some extreme values of standardized predicted values over the value 1 of the horizontal axis. The number of these values is mostly four of five, but there are eight values for Spain. We may assume that the linearity condition is fulfilled for all countries, but maybe not for Spain and the UK.

We can almost say the same about the heteroscedasticity of the residuals. Even there are extreme values on the right of each model, we cannot be sure that the heteroscedasticity is confirmed. This is because, even though the values scatter on the right side, but on the other hand they do not scatter equally, as on the left side.

#### Normality of the residuals

**Fig. 5:**
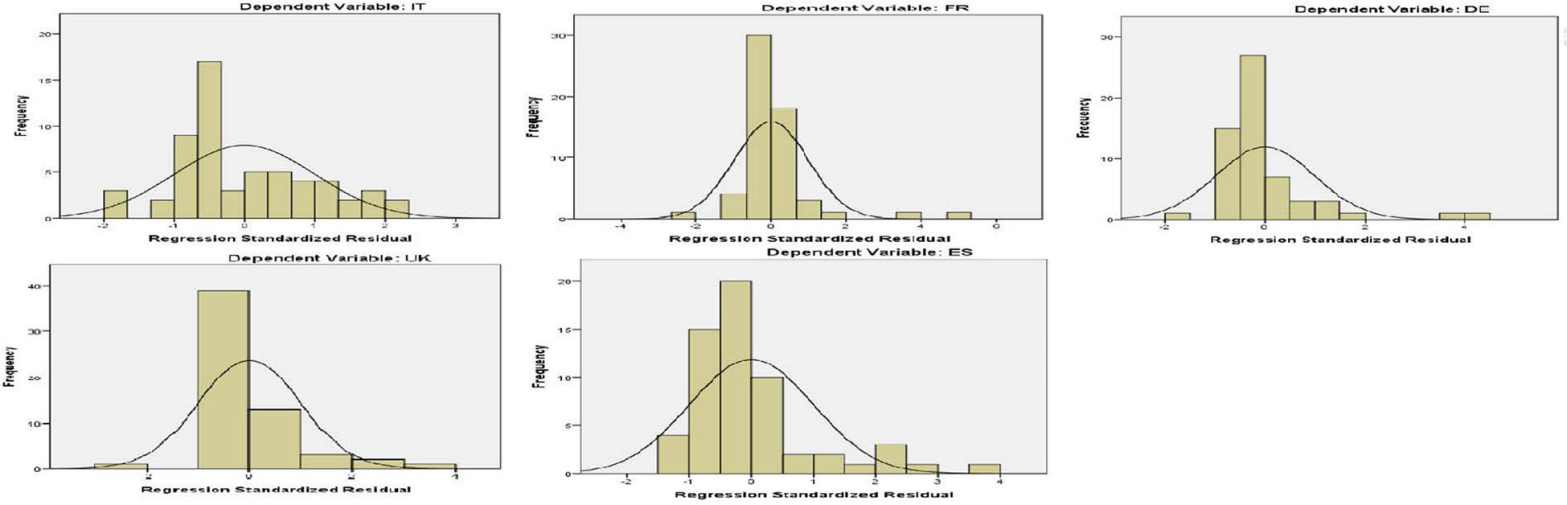
Histograms for residuals against normal distribution

We analyzed above the observations of Normal P-P plots for standard residuals. In this section we further proceed to some other tests to test the normality condition of the residuals. In the following Fig. 5 we present the histograms with the normality line.

From the above figure, we can see that the maximum frequency of the standard residuals is located close to the middle of the normal distribution line. Only in the first graph on the left for Italy, the maximum frequency is observed a little left from the center of the normal line.

**Fig. 6:**
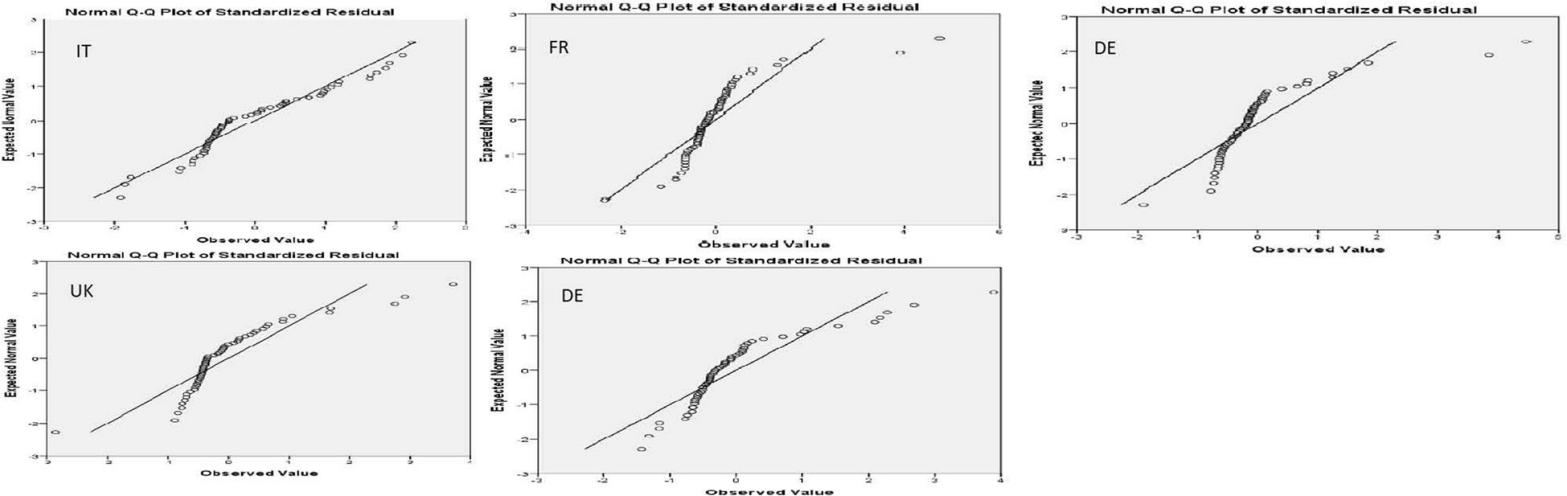
Normal Q-Q Plots for residuals

The normal Q-Q plots are shown in Fig. 6. Comparing to the normal P-P plots, we can see that for the UK and Spain, a considerable number of extreme values are observed away for the fitted line on the left. It is almost the same patter we noticed in the normal P-P plots. For Italy, the observed values are very close to the fitted line and for the rest of the countries, they gather close to line, but at larger distance than Italy.

Finally, the *Kolmogorov-Smirnov* and *Shapiro-Wilk* tests showed that the model is partially validated, since the condition of the normality of the residuals is not satisfied in all cases.

In Table 5, we can see the results of the Kolmogorov-Smirnov and Shapiro-Wilk tests for residuals. We can see that the combined results, as well as the results for the three first countries fulfill all criteria. In general, in our research, the residuals analysis did not reveal any significant pattern. As expected, the results show that this model is partly validated for all countries, but it was individually validated for Italy, France, Germany and partially for Spain.

**Table 5:** Kolmogorov-Smirnov and Shapiro-Wilk tests

country	test	***p***-value	condition satisfied	Validated
IT	Kolmogorov-Smirnov	< 0.001	yes	yes
IT	Shapiro-Wilk	0.005	yes	
FR	Kolmogorov-Smirnov	< 0.001	yes	yes
FR	Shapiro-Wilk	< 0.001	yes	
DE	Kolmogorov-Smirnov	< 0.001	yes	yes
DE	Shapiro-Wilk	< 0.001	yes	
UK	Kolmogorov-Smirnov	0.086	no	no
UK	Shapiro-Wilk	0.002	yes	
ES	Kolmogorov-Smirnov	< 0.001	yes	yes
ES	Shapiro-Wilk	< 0.001	yes	
all countries	Kolmogorov-Smirnov	0.076	no	no
all countries	Shapiro-Wilk	0.191	no	

**Fig. 7:**
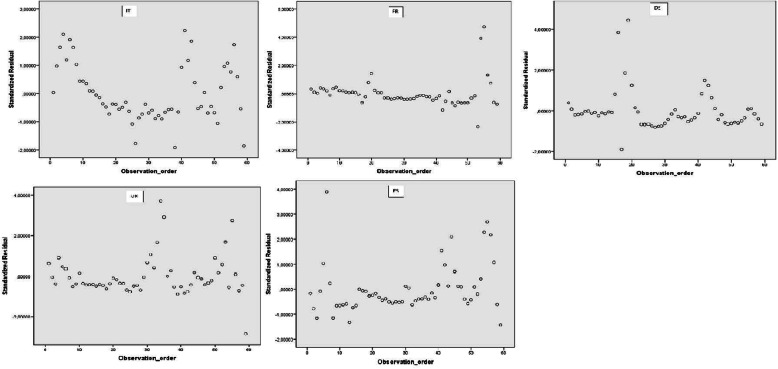
Standard residuals versus order

The same can be said about the analysis versus order. In the following Fig. 7 we can see that scatterplots do not reveal a specific pattern upwards or downwards, as the residuals bounce randomly around the residual=0 line. The final evaluation of the defined criteria can be summarized as in the following Table 6.

**Table 6:** Summary of evaluation criteria

c	Criterion description	Value	all	IT	FR	GE	UK	SP
1	Correlation: > 0.650 and p< 0.01	Yes	√	√	√	√	√	
2	Time accuracy of outbreak (months)	≤ 1	√	√	√	√		
3	Accuracy of high values: difference (%)	≤ 28%	√	√	√	√		√
4	Prediction of all high values	Yes	√	√	√	√	√	√
5	MSE (%)	< 28%	√	√	√	√	√	
	**Total**		**5**	**5**	**5**	**5**	**3**	**2**

#### Time-series model test

**Fig. 8:**
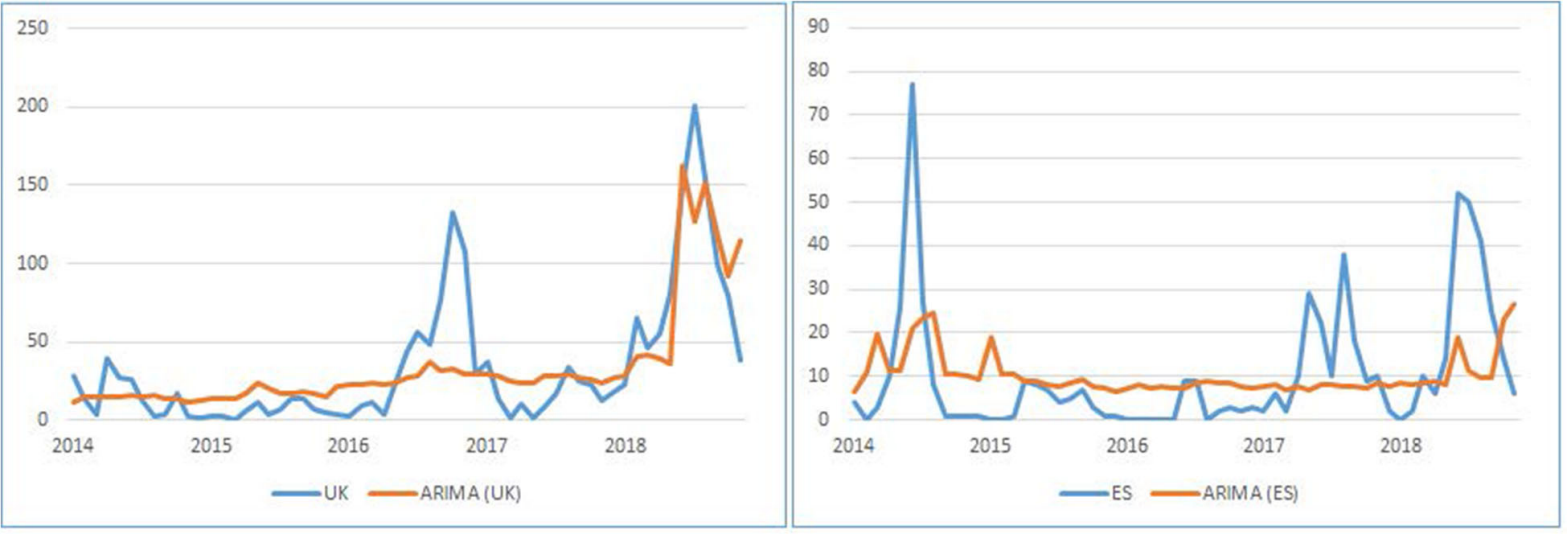
ARIMA model for the UK and Spain

The results show that the ARIMAX model cannot produce significantly better estimation compared to the SLR model. In the following Fig. 8, we present the predicted measles cases against the real values.

Comparing the two models with the SLR models, we see that ARIMAX prediction is almost on par with those of the SLR with a very little improvement. In the following Table 7 we can see the comparison results.

**Table 7:** Comparison of SLR and ARIMA

no	Description	SLR (UK)	ARIMA (UK)	SLR (ES)	ARIMA (ES)
1	Correlation:	0.785	0.781	0.322	0.326
2	Significance	< 0.001	< 0.001	0.011	0.011
3	MSE (%)	26.90	26.88	15.7	14.43

## Discussion

### Epidemiology and forecast

The described results show the potential of using search engine data to help predict the measles development and its spread in the countries of the EU/EEA with the largest population. The overall prediction model shows statistically significant correlation between measles cases and predicted values. Despite some deviations in time and in volume, the measles outbreaks are captured well through the suggested prediction models. On the other hand, significant differences occur among the examined five countries. As a general rule, countries with a relatively low activity of measles show less efficient prediction, such as Spain and secondly the UK. Another reason that these deviations may occur, is that Google search volume contains a sample of all searches made by people. This may introduce a small degree of error only for common search terms, and searches with a low overall volume can produce more variable results. This, of course, does not mean that we cannot predict the high activity on these countries, but the predictions have less statistical significance and less accuracy.

Comparing measles in the EU/EEA with the US is possible, since the population of the examined large countries and the US are equivalent. The largest five countries of the EU/EEA represent 323,256,460 population estimated for 2018 and the US has 328,863,150 (est. 2018) [26]. However, it seems that measles is a greater problem in Europe than in the US during the period from 2010 to 2016. The annual average of cases in the five countries during these years is 1306.60, while in the US the annual average reaches 209.57 cases, according to CDC data and their last report. That means that in the countries of the EU/EEA the problem is bigger, over six times more for the period of 2010–2016. France only, in 2011 reported 15,206 cases in the European health agency (ECDC) and CDC noticed that, during this year, most cases in the US were imported from France by travelling. Bearing in mind that the financial burden in the US was annually calculated over 200 million US$, the capability of successful monitoring and predicting measles in the EU/EEA becomes too important, if not critical.

Another aspect is the early prediction. ECDC publishes reports monthly, but it includes data on measles 2 or 3 months prior to every month of the report announcement. For instance, the data for the entire year of 2016 (from January to December) were announced in the report of 7 Apr 2017 [27]. By obtaining data from Google Trends and building prediction patterns and models, we achieve an early prediction at least 2 months in advance. According to the US Centers of Disease Control and Prevention (CDC, 2004) [28], syndromic surveillance has been used for early detection of outbreaks*.* Its purpose is to follow the size, spread, and tempo of outbreaks, to monitor disease trends, and to provide reassurance that an outbreak has not occurred. Under this vision, Internet data has the potential to help in this purpose.

The nature of *Epidemiology* is essential, as it tries to find out the association of cause and effect, regarding epidemics [29]. In this research, we attempted to find the association of measles and public interest, as it is expressed through Internet queries over time. The contribution of our study can be summarized as follows: i) It extends the limited literature on prediction of measles, not through epidemiological data, but rather from Internet data. Measles is a disease that has not been examined as much as other epidemic diseases, such as influenza, dengue, AIDS/HIV, Ebola, etc. ii) There are also some comparative studies, but not too many. Therefore, in our work we focused on presenting comparative results between different countries for an extended period of 5 years and finally iii) this work classifies and constructs quantitative evaluation criteria, based on specific measurements to test the requirements of the proposed models to be used. In previous studies, no matter which model is used, all research has been conducted with a measurement of correlation coefficients only.

### Limitations

There are certain limitations, concerning both Google Trends data and the use of Python. As previously mentioned, prediction models can have major deviations between the examined five countries. In case of a relatively low activity of measles, under the mean of 370.5 cases per month, predictions may not be as good for some countries, such as the UK and Spain by just using a single regression model. The alternative is to produce further models, based on Google data from other countries and from these countries with low activity of measles, by using weighted estimations. Such techniques may facilitate the improvement of the forecast.

Python itself has its own limitations. Pytrends API works, but we initially found difficulty to use terms corresponding to the Spanish language. “*Sarampión”*, the equivalent of the term *measles*, when used to get the data from Google Trends, gives data with too many zeros. The alternative was to use the same word without the specific Spanish spelling, but as “*Sarampion”.* Another issue is the overlapping period we use to address the maximum time limit of acquiring data from the past. We successfully dealt with this problem by using overlapping period of 250 days.

Data from Internet that are not epidemiological can certainly achieve precise predictions through search engines or other sources, such as social media. Nevertheless, it is common that sometimes, these estimations or forecasts are not 100% precise, as other similar studies show. For instance, in the work of E. Oren et al. (2018), mentioned in the Introduction [12], correlation coefficients for influenza vary from 0.46 to 0.72 for all search terms from the Internet for different States of the US. O.E. Santangelo et al. (2019) [30] examine measles in Italy and by correlating weekly data, they find that correlation varies for different lags of weeks, from 0. 6152 to 0.70 and 0.8271. In addition, in some periods, these estimations are either underestimated or overestimated. This might happen because people do not equally react to real cases of life; sometimes they exaggerate real situations or even express less interest in them, especially on health issues. It is the human nature that causes this effect. Another issue is that some data from Google may cause less precision in prediction or heteroscedastic issues. The latter is not critical [31] as it does not affect the estimated values of the intercept or slope coefficients of a linear regression model. On the other hand, it has affection on the estimated standard errors for those coefficients. This means that it can make them either too large or too small. This inadequacy of the data may result to a worse prediction of epidemics, even in the case that another model is used, e.g., a time-series model, such as ARIMA. It is possible though to use data from Internet to establish rules and patterns of epidemics by taking advantage of non-epidemiological data in a successful way for monitoring and preventing purposes.

## Conclusions

The spread of measles differs between the largest countries of the EU/EEA. What they have in common is that outbreaks always occur in the first quarter of the year, but not every year, usually every March or April. The financial costs for measles are substantial regarding treatment and hospitalization that are needed due to the implications caused by the infection. Although there are no official aggregated costs for Europe, but only for some countries [32], it is certain that exceed the corresponding expenditure of the US; maybe over 1 billion US$ (5 years). Comparing to the US, in Europe the problem of measles seems to be greater.

Data from Google Trends can help in tracking the spread, the size and the development of measles in a relatively robust and accurate way. Linear regression models can produce good estimations. Countries with a relatively low activity or impact of measles may show problems in prediction of measles cases. In these cases, alternative techniques can be applied, combining data from other countries. Nevertheless, in these cases, despite the application of those enhancing techniques, it is not always feasible to produce reliable predictions.

By using Python and the Pytrends API, data can be obtained from Google Trends, despite some restrictions and limitations. These data can be gathered in an inexpensive way in terms of computing resources.

## Data Availability

The epidemiological data can be directly found through the ECDC website: https://www.ecdc.europa.eu/en/measles/surveillance-and-disease-data/monthly-measles-rubella-monitoring-reports). Data from Google Tends are available with the installation and use of the Pytrends API with the proper parameters (country, search terms written in the desired language, etc.). The latest version of this API (version 4.7.3.) can be obtained from the official website https://pypi.org/project/pytrends/

## Abbreviations

AIDS: (Human Immunodeficiency Virus infection and) Acquired Immune Deficiency Syndrome (HIV/AIDS)
API: Application Programming Interface
ARIMAX: Auto-Regressive Integrated Moving |Average model (with exogenous variable)
CDC: Centers for Disease Prevention and Control
CPU: Central processing Unit
DE: Germany
ECDC: European Centre for Disease Prevention and Control
ES: Spain
EU/EEA: European Union/ European Economic Area
FR: France
IBM SPSS: Statistical Package for the Social Sciences
IT: Italy
ISO: International Standardization Organization
MD: Mean Difference
MSE: Mean Standard Error
MMR: Measles, Mumps, and Rubella
OS: Operational System
RAM: Random Access Memory
RQ: Research questions
SLR: Simple Linear Regression
UK: United Kingdom
US: United States (of America)
US$: United States Dollar
WHO: World Health Organization
